# Prognosis of PD-L1 in human breast cancer: protocol for a systematic review and meta-analysis

**DOI:** 10.1186/s13643-020-01306-9

**Published:** 2020-03-26

**Authors:** Isnard Elman Litvin, Machline Paim Paganella, Eliana Marcia Wendland, Adriana Vial Roehe

**Affiliations:** 1 Graduate Program in Pathology, Federal University of Health Sciences of Porto Alegre, Porto Alegre, RS Brazil; 2 School of Medicine, University of Caxias do Sul –UCS, Caxias do Sul, RS Brazil; 3 Instituto de Pesquisas em Saúde (IPS), University of Caxias do Sul –UCS, Caxias do Sul, RS Brazil; 4 Federal University of Health Sciences of Porto Alegre, Community Health Department, Porto Alegre, RS Brazil

**Keywords:** PD-L1, Human Breast Cancer, Systematic Review, Meta-analysis, Protocol

## Abstract

**Background:**

Breast cancer is one of the most common malignancies in women worldwide, and one of the leading causes of cancer-related death. Programmed cell death 1 (PD-1) and its ligand (PD-L1) are key physiologic suppressors of the cytotoxic immune reaction. Some authors advocate that PD-L1 expression may help in breast cancer prognosis.

**Methods:**

We will conduct a systematic review of observational or interventional studies evaluating the prognostic ability of PD-L1 expression levels in predicting positive clinical outcomes in Human Breast Cancer. A sensitive search strategy will be employed in MEDLINE, EMBASE, LILACS, The Grey Literature Report, OpenGrey, OAIster, and Cochrane CENTRAL. Two reviewers will independently screen all identified references for eligibility and extract data. The outcomes evaluated will be Overall Survival, Breast Cancer-specific Survival, Disease-free Survival, Recurrence-free Survival, Positive Lymph Node, and Distant Metastasis. The outcomes will be extracted directly from the studies, if available. Methodological quality and bias of included studies will be assessed using a standardized checklist and overall quality of evidence will be assessed through the Grading of Recommendations Assessment, Development and Evaluation (GRADE) approach. If meta-analysis is possible, the measures of association will be calculated using bivariate random-effects models. Statistical heterogeneity will be evaluated with *I*^2^ statistics and explored through sensitivity analysis.

**Discussion:**

Immunomodulation seems to be a promising strategy in solid tumors. Breast cancer is the most common malignancies in women worldwide, and one of the leading causes of cancer death. PD-1 and PD-L1 are key physiologic suppressors of the cytotoxic immune reaction.

**Trial registration:**

Systematic review registration: CRD42019121118 (PROSPERO)

## Background

Breast cancer (BC) is one of the most common malignancies in women worldwide, and one of the leading causes of cancer death [1, 2]. In BC, the bulk of evidence showed that immune cells infiltration presented in the tumor, especially tumor-infiltrating lymphocytes (TILs), were associated with clinical outcomes in some malignant tumors [2–6]. TILs evaluated in primary BC may convey prognostic information [7], although their precise use remains unclear [8].

The programmed cell death-1 receptor (PD-1) is an immune checkpoint inhibitor that is expressed on the surface of immune effectors cells. It is activated mainly by its ligand (PD-L1) which can be expressed by all human cells. The PD-1/PD-L1 pathway plays a subtle role in maintaining peripheral T-lymphocyte tolerance and regulating inflammation [9].

PD-1 and PD-L1 are key physiologic suppressors of the cytotoxic immune reaction. Results from preclinical studies support the idea that inhibition of PD-L1 and PD-1 axis in the tumor microenvironment may promote tumor regression, and in clinical trials, various agents targeting PD-1 or PD-L1 have demonstrated robust response rates in a variety of tumor types [10–16]. Limited data have been reported on the expression of PD-L1 in tumor cells and/or immune cells in breast cancer, but preliminary reports are divergent [16–20]. Few studies have evaluated the expression of the programmed cell death-1 and its ligand-1 (PD-L1) in breast cancer [21].

Hou et al. evaluated PD-L1 and a set of other relevant immune markers in relation to their association with clinical outcome in a series of HER2-positive BC cases. They suggest that cytotoxic immune reaction mediated by CD8-positive T cells and PD-L1 expression may predict a better outcome in patients with HER2-positive BC managed with conventional chemotherapy and HER2-blocking therapy [16].

The purpose of this protocol of systematic review and meta-analysis is to assess the prognostic value of PD-L1 in Human Breast Cancer.

## Methods

### Protocol and registration

The protocol for this review was defined a priori and registered online in the PROSPERO international prospective register of systematic reviews (CRD42019121118). The methodological approach to evidence searching and synthesis, described in this protocol, will follow the Preferred Reporting Items for Systematic Review and Meta-Analysis Protocols (PRISMA-P) recommendations [22] (see Additional file 1) and MOOSE Guidelines for Meta-Analyses and Systematic Reviews of Observational Studies [23].

### PECO question

For systematic review and meta-analysis of studies on risk factors and prognosis, the acronym PECO is used, which corresponds to the areas P (population), E (exposure), C (comparison) and O (outcome). The STEEP system was considered as a standardized definition system of the outcomes [24]. The formulated PECO question is: What is the prognostic significance of programmed cell death 1 (PD-L1) expression in breast cancer?

The definitions for the acronym PECO for this systematic review are the following:

- Population: women aged 18 years or more with breast cancer pathologically confirmed of any type
- Exposure: PD-L1-positive based on immunohistochemistry (IHC)
- Comparison: PD-L1-negative based on IHC
- Outcomes (and its definitions):
  - ○ Overall survival (OS): death from breast cancer, death from a non-breast cancer cause, and death from unknown cause
  - ○ Progression-free survival (PFS)
  - ○ BC-specific survival (BCSS)
  - ○ Disease-free survival (DFS): invasive ipsilateral breast tumor recurrence, local/regional invasive recurrence, distant recurrence, death from breast cancer, death from a non-breast cancer cause, death from an unknown cause, invasive contralateral breast cancer, and second primary invasive cancer (non-breast)
  - ○ Recurrence-free survival (RFS): invasive ipsilateral breast tumor recurrence, local/regional invasive recurrence, distant recurrence, death from breast cancer, death from a non-breast cancer cause, and death from an unknown cause
  - ○ Positive lymph node (PLN)
  - ○ Distant metastasis (DM)
  - ○ Distant disease-free survival (DDFS): distant recurrence, death from breast cancer, death from a non-breast cancer cause, death from an unknown cause, second primary invasive cancer (non-breast)
  - ○ Pathological response after neoadjuvant chemotherapy

### Criteria for considering studies for this review

In the review, we will include any observational (cohort) or intervention studies (randomized controlled trials) which evaluate the prognostic ability of PD-L1 expression using IHC (using any method and any type of PD-L1 clone) in women with breast cancer. We will include studies that evaluate PD-L1 positivity with different cut-offs according to various scoring systems including Histo-score system (H-score), PD-L1 expression in tumor/normal breast samples (T/NB ratio), 4-point scale, Allred score, or Immunoscore (staining intensity and percentage of PD-L1 positive tumor cells). When studies include both women with and without breast cancer, only women with the target disease will be evaluated. Articles will be excluded from the analyses based on the following criteria: PD-L1 expressed only on other cells (e.g., immune cell and stromal cell), not tumor cell; non-human experiments, case reports, case series, case-control, animal testing, narrative reviews, duplicate publication, meeting abstracts, and expert opinions. Studies that are published as abstract only and whose detailed information cannot be obtained from the authors will be excluded.

### Search methods for identification of studies

The keywords and Medical Subject Headings related to PD-1, PD-L1, and Human Breast Cancer will be used alone or in combination (and with synonyms and closely related words) in order to retrieve relevant articles.

We will search in Excerpta Medica Database (EMBASE), Centro Latinoamericano y del Caribe de Información en Ciencias de la Salud (LILACS), Medical Literature Analysis and Retrieval System Online (MEDLINE), Cochrane Central Register of Controlled Trials (CENTRAL), and Web of Science. We will also screen reference lists of relevant studies and reviews for additional articles and will search websites of Grey Literature such as The Grey Literature Report, OpenGrey and Open Archives Initiative (OAIster). If necessary (unclear data, missing data, and extractable data). The search strategies developed for each database are shown on Additional file 2. There will be no language or publication year restriction. The PRISMA Flow Diagram will be used to depict the flow of information through the different phases of a systematic review (see Additional file 3).

### Study selection process

Potentially eligible studies will be screened for inclusion eligibility independently by two review authors (IEL and AVR) based on their title, abstract, and full text. A third author (MPP) will adjudicate any discrepancies. Reasons for excluding full-text studies will be documented.

### Data collection

From eligible studies, we will extract bibliographical and study description data (e.g., title, author, country, study design, language of publication, year of publication, sample size, number of centers), patient characteristics (e.g., total number and number in comparison groups, age), and data related to breast cancer and prognosis. Data collection will be performed, independently and in duplicates by two reviewers (IEL, AVR), using predefined data fields (Table 1). Table 1 will be pilot-tested to assess for usability and any amendments will be performed if required. We will attempt to establish contact with the corresponding authors for missing data.

**Table 1:** Characteristics of the studies included

#	Title	Author	Year	Country	Language	Study design	Age	# centers	Median follow-up	Sample size	Stage	Treatment	Breast cancer subtype–IHC	PD-L1				Outcomes/end points						
														Clone	+	-	%							
1																								
2																								
3																								
4																								

Breast cancer subtypes–immunohistochemistry: luminal A, luminal B, HER2-positive or triple-negative

Outcomes: *OS* Overall survival: death from breast cancer, death from non-breast cancer cause and death from unknown cause, *PFS* progression-free survival

*BCSS* BC-specific survival, DFS disease-free survival: invasive ipsilateral breast tumor recurrence, local/regional invasive recurrence, distant recurrence, death from breast cancer, death from non-breast cancer cause, death from unknown cause, invasive contralateral breast cancer, and second primary invasive cancer (non-breast), *RFS* recurrence-free survival: invasive ipsilateral breast tumor recurrence, local/regional invasive recurrence, distant recurrence, death from breast cancer, death from non-breast cancer cause, and death from unknown cause, *PLN* positive lymph node, *DM* distant metastasis, DDFS distant disease-free survival: distant recurrence, death from breast cancer, death from non-breast cancer cause, death from unknown cause, and second primary invasive cancer (non-breast)

Pathological response after neoadjuvant chemotherapy: Stage: I, II, III, IV. Treatment: surgery status, neoadjuvant chemotherapy status, adjuvant endocrine and chemotherapy status, and radiotherapy

### Risk of bias assessment

Quality In Prognosis Studies (QUIPS) tool will be used to assess the risk of bias in prognostic factor studies [25]. The QUIPS tool rates six bias domains: study participation, study attrition, prognostic factor measurement, outcome measurement, study confounding, and statistical analysis and reporting, as having a high, moderate, or low risk of bias [26]. The Grading of Recommendations Assessment, Development, and Evaluation (GRADE) will be used to rate the quality of the body of evidence retrieved [27, 28].

### Statistical analysis

If identified as possible (the studies retrieved have quantitative data reported that can be combined), the extracted data will be aggregated into a meta-analysis by "R" Software. Hazard ratios (HRs) and 95% confidence intervals will be pooled to measure the time to event relationship (between the potential prognostic factor and tumor recurrence). Data derived from the multivariate analysis will be used as default, but when absent, univariate values will be used. A combination of adjusted and unadjusted hazard ratio estimates for the association between PD-L1 expression and breast cancer will be managed by using the patient-level correlation as an approximation for the within-study correlation [29]. Results from interventional and from observational studies will be pooled separately, in a sensitivity analysis. Standard errors will be calculated from confidence intervals and the individual study associations [30–33]. The measure of association estimates will be weighted and combined using the generic inverse variance and random effect model.

To allow the readers to visualize any general trends or mixed findings across the studies, we intend (if possible) to present a Forest Plot with the individual study estimates and confidence intervals.

Publication bias will be assessed by visual inspection of the funnel plot or Egger’s test, according to the number of articles included. Heterogeneity will be assessed by the Cochran Q and *I*^2^ statistics. When more than one threshold is available, all data will be recorded. Sensitivity analysis will be conducted to assess the impact of including studies with 20% or more of missing data and also to study the impact of the different study designs. The impact of study design (cohort versus clinical trials) will be evaluated in a sensitivity analysis. Subgroup analysis will be performed, if possible regarding the KI-67 index, Tumor Size and Type, Nottingham Grade, ER/PR (Estrogen Receptor/Progesterone Receptor) Status and IHC method/clone type. All statistical tests will be 2-sided, and statistical significance will be defined as *p* < 0.05.

## Discussion

High level of immune infiltration in the primary tumor, measured by the number of TILs or immune gene expression signatures, has been associated with longer survival and response to neoadjuvant chemotherapy in triple-negative and HER2 positive breast cancers (not in luminal A breast cancers) [34–43]. A strong lymphocytic infiltration in the residual tumor after neoadjuvant chemotherapy has also been associated with longer survival. The immune microenvironment influences the efficacy of chemotherapy and radiotherapy; these treatments cause an immunogenic death of the malignant cells and/or somatic mutations leading to neoantigens that elicit an adaptive immune response which will clear or keep the escaping tumor cells dormant [43, 44].

PD-1/PD-L1 inhibitors have shown promising activity in the first clinical trials in breast cancer, and some trials are testing their efficacy and safety in the metastatic and neoadjuvant setting [43, 45, 46].

Immunomodulation seems to be a promising strategy in solid tumors where the PD-1/PD-L1 inhibitory pathway can be misused to silence the immune system by increasing the expression of PD-L1 on the tumor cell surface. PD-L1 expression has been associated with: large tumor size, high grade, high proliferation, estrogen receptor-negative status, and HER2-positive status, and it is inversely correlated with survival in breast cancer [9, 18, 47]. According to Mittendorf et al., PD-L1 is expressed in 20% of triple-negative breast cancers (TNBCs) [20].

Muenst et al. published the first study to demonstrate that PD-L1 expression is an independent negative prognostic factor in human breast cancer. They conducted an IHC study using a tissue microarray encompassing 650 evaluable formalin-fixed breast cancer sample cases with detailed clinical annotation and outcomes data. PD-L1 was expressed in 152 (23.4%) of the 650 breast cancer specimens. Expression was significantly associated with age, tumor size, AJCC (American Joint Committee on Cancer) primary tumor classification, tumor grade, lymph node status, absence of ER expression, and high Ki-67 expression. In univariate analysis, PD-L1 expression was associated with a significantly worse overall survivor [48].

Sabatier et al. retrospectively analyzed PD-L1 mRNA expression in 45 breast cancer cell lines and profile 5454 breast cancers using DNA microarrays. They found that compared to normal breast samples, PDL1 expression was upregulated in 20% of clinical samples and 38% of basal tumors and concluded that PD-L1 upregulation was associated with increased T cell cytotoxic immune response. In this aggressive subtype, upregulation was associated with better survival and response to chemotherapy [18].

A previous meta-analysis performed by Wang et al. supported the notion that high PD-L1 expression in breast cancer is correlated with poor prognosis. Their study was restricted to English publications and included PD-L1 assessed by mRNA level and IHC [49].

Many issues remain to be answered, such as the identification of biomarkers able to predict for a clinical benefit of PD-L1 inhibitors. This systematic review can help to clarify the prognostic significance of PD-L1 expression in breast cancer.

## Supplementary information

**Additional file 1:.** PRISMA-P.

**Additional file 2:.** Database searches.

**Additional file 3:.** PRISMA Flow Diagram.

## Data Availability

Not applicable.

## Abbreviations

AJCC: American Joint Committee on Cancer
BC: Breast cancer
BCSS: Breast cancer-specific survival
CENTRAL: Cochrane Central Register of Controlled Trials
DDFS: Distant disease-free survival
DFS: Disease-free survival
DM: Distant metastasis
EMBASE: Excerpta Medica Database
ER: Estrogen receptor
GRADE: Grading of Recommendations Assessment, Development and Evaluation
HR: Hazard ratio
IHC: Immunohistochemistry
LILACS: Centro Latinoamericano y del Caribe de Información en Ciencias de la Salud
MEDLINE: Medical Literature Analysis and Retrieval System Online
MOOSE: Meta-analyses Of Observational Studies in Epidemiology
OAIster: Open Archives Initiative
OS: Overall survival
PD-1: Programmed cell death-1 receptor
PD-L1: Programmed cell death-1 receptor ligand 1
PECO: Population, Exposure, Comparison, Outcome
PFS: Progression-free survival
PLN: Positive lymph node
PR: Progesterone receptor
PRISMA: Preferred Reporting Items for Systematic Review and Meta-Analysis
PRISMA-P: Preferred Reporting Items for Systematic Review and Meta-Analysis Protocols
QUIPS: Quality In Prognosis Studies
RFS: Recurrence-free survival
TILs: Tumor-infiltrating lymphocytes
