# Supplementary material for: Prognosis of PD-L1 in human breast cancer: protocol for a systematic review and meta-analysis
Source: Syst Rev. 2020 Mar 26;9:66. doi: 10.1186/s13643-020-01306-9 (PMC7098137; doi:10.1186/s13643-020-01306-9)
Supplement: Supplementary file 2 — Additional file 2:. Database searches. [file 13643_2020_1306_MOESM2_ESM.docx]

ADDITIONAL FILE 1

| Database | Search Strategy |
| --- | --- |
| PUBMED | ((("humans"[MeSH Terms] OR "humans"[All Fields] OR "human"[All Fields]) AND ("breast neoplasms"[MeSH Terms] OR ("breast"[All Fields] AND "neoplasms"[All Fields]) OR "breast neoplasms"[All Fields] OR ("breast"[All Fields] AND "cancer"[All Fields]) OR "breast cancer"[All Fields])) AND (PD-1[All Fields] OR PD-L1[All Fields])) AND (((((("mortality"[Subheading] OR "mortality"[All Fields] OR "survival"[All Fields] OR "survival"[MeSH Terms]) OR ("progression-free survival"[MeSH Terms] OR ("progression-free"[All Fields] AND "survival"[All Fields]) OR "progression-free survival"[All Fields] OR ("progression"[All Fields] AND "free"[All Fields] AND "survival"[All Fields]) OR "progression free survival"[All Fields])) OR (("breast neoplasms"[MeSH Terms] OR ("breast"[All Fields] AND "neoplasms"[All Fields]) OR "breast neoplasms"[All Fields] OR ("breast"[All Fields] AND "cancer"[All Fields]) OR "breast cancer"[All Fields]) AND specific[All Fields] AND ("mortality"[Subheading] OR "mortality"[All Fields] OR "survival"[All Fields] OR "survival"[MeSH Terms]))) OR (("recurrence"[MeSH Terms] OR "recurrence"[All Fields]) AND free[All Fields] AND ("mortality"[Subheading] OR "mortality"[All Fields] OR "survival"[All Fields] OR "survival"[MeSH Terms]))) OR (positivie[All Fields] AND ("lymph nodes"[MeSH Terms] OR ("lymph"[All Fields] AND "nodes"[All Fields]) OR "lymph nodes"[All Fields] OR ("lymph"[All Fields] AND "node"[All Fields]) OR "lymph node"[All Fields]))) OR (distant[All Fields] AND ("neoplasm metastasis"[MeSH Terms] OR ("neoplasm"[All Fields] AND "metastasis"[All Fields]) OR "neoplasm metastasis"[All Fields] OR "metastasis"[All Fields]))) |
| EMBASE | ('human breast cancer' OR (('human'/exp OR human) AND ('breast'/exp OR breast) AND ('cancer'/exp OR cancer))) AND ('pd 1' OR 'pd l1') AND ('overall survival' OR 'progression free survival' OR 'breast cancer specific survival' OR 'recurrence free survival' OR 'positive lymph node' OR 'distant metastasis') |
| Web of Science | "human breast cancer" AND (pd-1 OR pd-l1) AND ("overall survival" OR "progression free survival" OR "breast cancer specific survival" OR "recurrence free survival" OR "positive lymph node" OR "distant metastasis") |
| LILACS | "human breast cancer" AND (pd-1 OR pd-l1) AND ("overall survival" OR "progression free survival" OR "breast cancer specific survival" OR "recurrence free survival" OR "positive lymph node" OR "distant metastasis") |
| Cochrane | ID Search Hits  #1 human breast cancer 14609  #2 pd-1 1327  #3 pd-l1 1384  #4 overall survival 44835  #5 progression free survival 22666  #6 breast cancer specific survival 1245  #7 recurrence free survival 9951  #8 positive lymph node 2730  #9 distant metastasis 2911  #10 #2 OR #3 2001  #11 #4 OR #5 OR #6 OR #7 OR #8 OR #9 54670  #12 #1 AND #10 AND #11 54 |
